# Infective Endocarditis Involving Mitral and Aortic Valves With Interatrial Septal Abscess

**DOI:** 10.7759/cureus.15730

**Published:** 2021-06-17

**Authors:** Jean Kim, James Ha, Christina Park, Eric Y Chung

**Affiliations:** 1 Internal Medicine, University of Hawaii, Honolulu, USA; 2 Cardiothoracic Surgery, The Queen's Medical Center, Honolulu, USA

**Keywords:** infectious endocarditis, valvular vegetation, intracardiac abscess, interatrial abscess, aortic valve, mitral valve

## Abstract

Infectious endocarditis (IE) is an infection of the endocardial surface and frequently refers to the infection of one or more heart valves. The clinical manifestations of IE are highly variable, with fever being the most common symptom, along with other nonspecific symptoms such as chills, anorexia, malaise, and myalgias. IE is associated with various systemic complications including septic emboli, cardiac complications such as valvular vegetations and intracardiac abscess, neurologic complications, and systemic immune reactions. In this case report, we present a patient with an IE that involved both mitral and aortic valves as well as a unique pathology with an interatrial septal abscess.

## Introduction

Infectious endocarditis (IE) is an infection of the endocardium that most commonly involves the cardiac valves. The incidence of endocarditis is approximately 15 per 100,000 person-years in the United States, and the incidence is on the rise [1]. Community-associated IE is the predominant form of the disease; however, there is an increasing number of cases attributable to IV drug use and in association with the increased use of cardiac implantable electrophysiological devices [1]. The clinical presentation of IE can be variable, but patients often present with flu-like symptoms, such as fever, malaise, and night sweats. Cardiac murmurs are present in about 85% of patients, while vascular phenomena such as Roth spots, Janeway lesions, and splinter hemorrhages are observed in fewer than 5% of cases [2]. IE can also be complicated by intracardiac pathologies, such as perivalvular abscess, pseudoaneurysm, or intracardiac fistulas, which could lead to heart failure, heart block, and even death if not treated appropriately. Most cases of IE involve one valve of the heart, and the involvement of multiple valves with interseptal abscess is a rare finding with no precise incidence being reported. In this case report, we present a case of a patient who presented with IE with rare pathology of both mitral and aortic valve involvement with interatrial septal abscess, which required multidisciplinary team management.

## Case presentation

**Figure 1:**
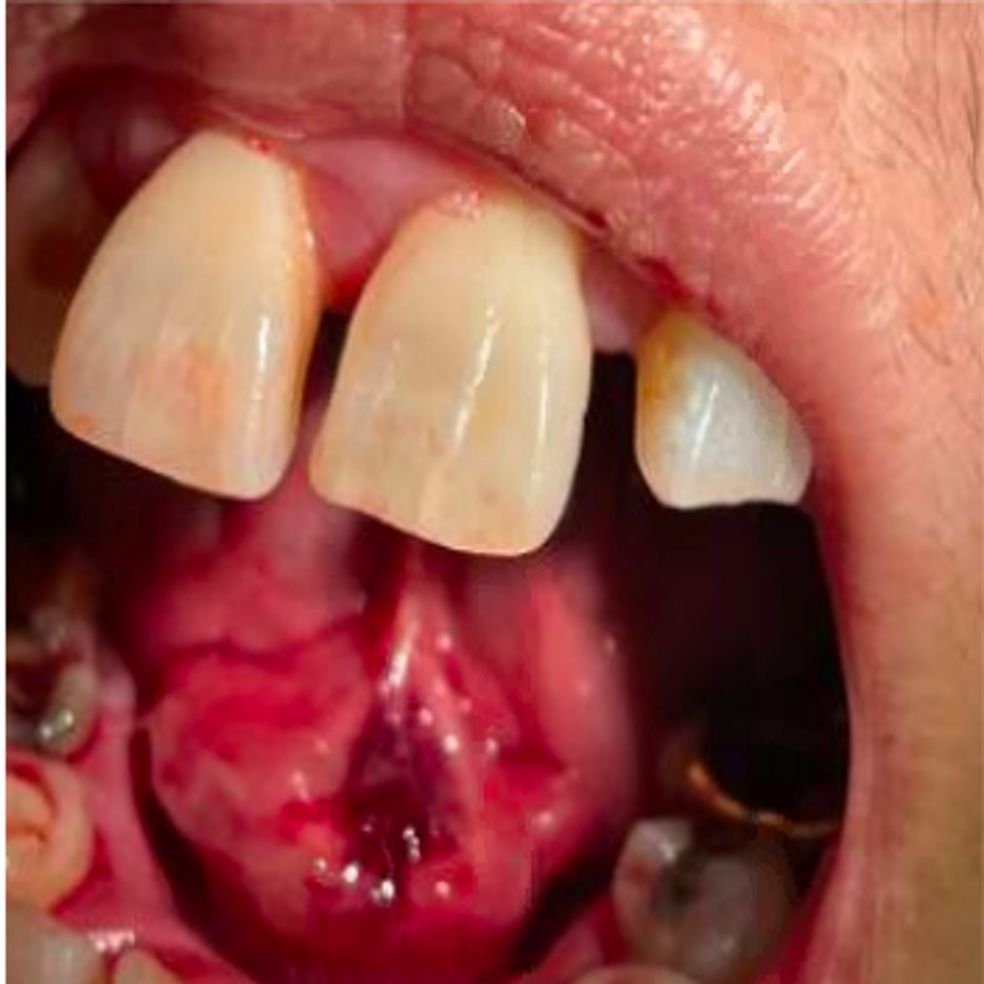
A fungating oral lesion with central ulceration and bleeding underneath the tongue.

A 60-year-old woman with a history of chronic back pain and bilateral sciatica presented with acute worsening of her low back pain for three days. The patient described her back pain as excruciating in severity, pinching in quality, and worse with movement. She also reported that the pain radiated down her bilateral legs, but denied lower-extremity weakness. The patient also reported that she recently noticed a painless bleeding oral lesion underneath her tongue. The patient denied recent facial or oral trauma, infection, or procedure. The patient also denied a history of smoking, alcohol, or illicit drug use. Vitals were notable for a temperature of 38.0°C, and the physical examination revealed poor dentition and a 2 x 1 cm fungating mass on the inferior aspect of the tongue with central ulceration and bleeding (Figure 1). The cardiac examination was notable for tachycardia with grade III/VI holosystolic murmur over the apex. Tenderness to palpation was elicited over lumbar spinous processes and paraspinal muscles. The neurologic examination was unremarkable with normal, equal strength and sensation throughout. 

**Figure 2:**
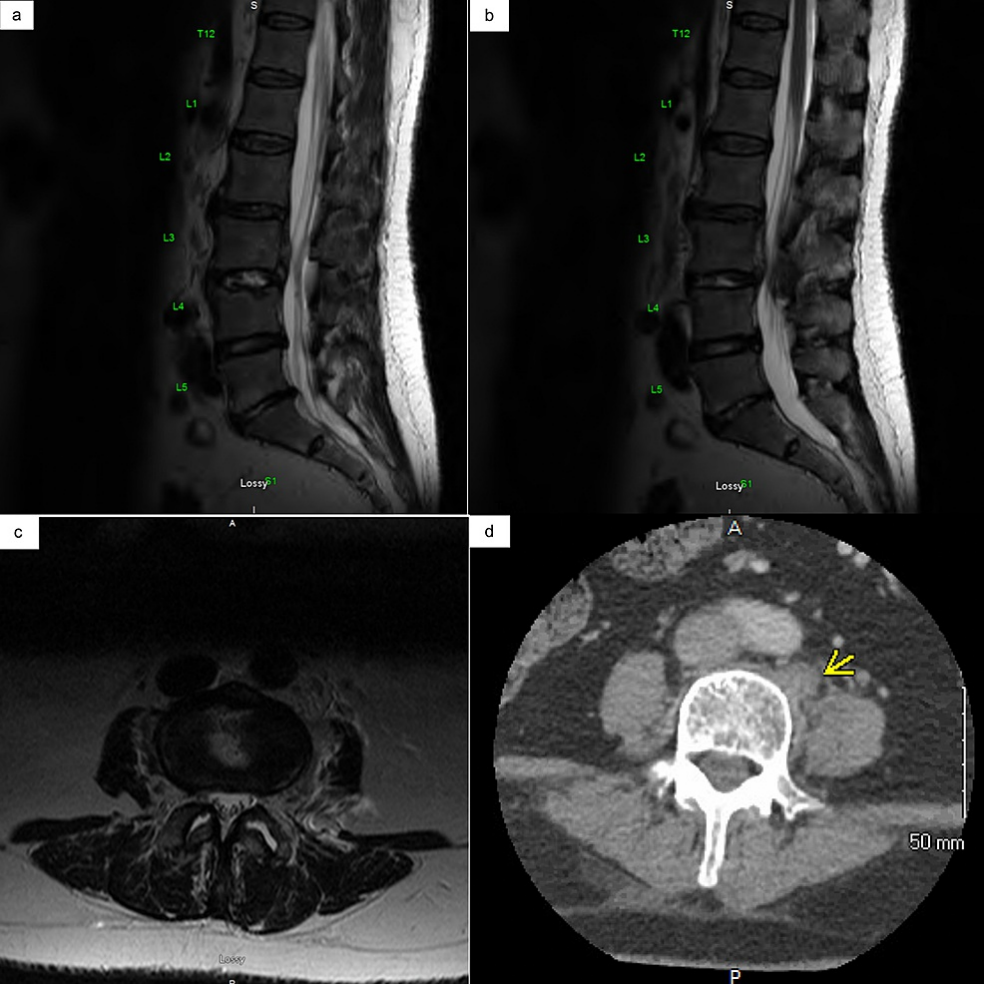
Lumbar spine MRI (a, b, c) and CT (d) with contrast. (a) Marked T2 hyperintense signal throughout the L3-4 intervertebral disc suggestive of edema and inflammatory change. (b) Abnormal epidural inflammatory changes extending superiorly from the L3-4 disc along the posterior margin of L3 vertebral body. Mild-to-moderate central spinal stenosis at and above the level of the L3-4 disc. (c) T2 hyperintense signal within the left psoas muscle suggestive of pyomyositis. (d) A prominent paraspinal soft tissue density along the anterolateral aspect of L4 vertebral body that may represent a phlegmon.

Labs were notable for an elevated white blood cell count of 16.56 x 10^3^/µL, erythrocyte sedimentation rate 83 mm/h, and C-reactive protein of 217 mg/L. Liver function test, renal profile, and cardiac biomarkers were within normal limits. The ECG was consistent with normal sinus rhythm. CT lumbar spine showed prominent paraspinal soft tissue density along the anterolateral aspect of L4 vertebral body that may represent a phlegmon (Figure 2). MRI of the lumbar spine showed an abnormal heterogeneous T2 hyperintense signal throughout the L3-4 intervertebral disc from edema and inflammatory change, suspicious for acute L3-4 discitis, vertebral body osteomyelitis, and paraspinal soft tissue infection (Figure 2).

**Figure 3:**
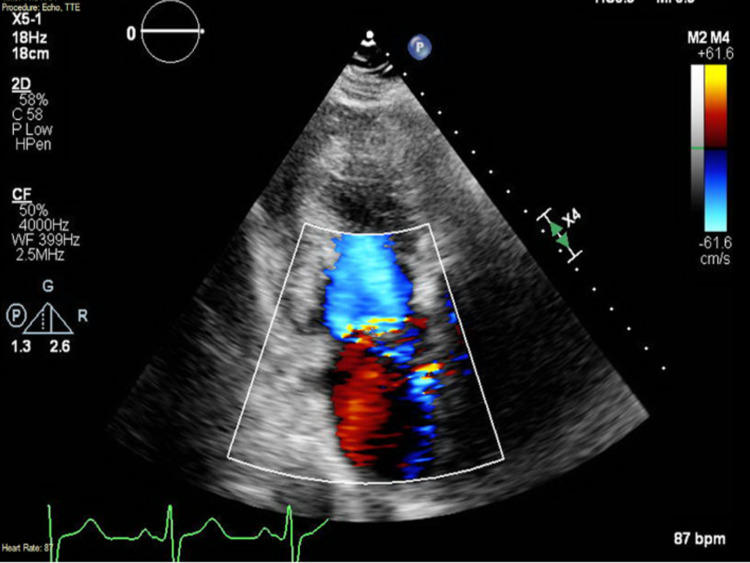
Transthoracic echocardiogram with two-chamber apical view showing anteriorly directed mitral regurgitation.

**Figure 4:**
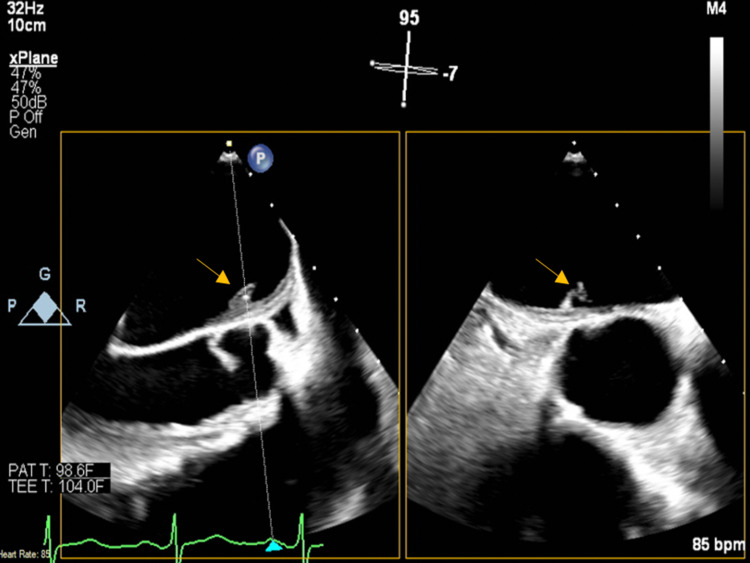
Transesophageal echocardiogram showing a small linear echodensity on the aortic valve.

Blood cultures grew *Streptococcus sanguinis* in two of two bottles. The transthoracic echocardiogram demonstrated severe mitral regurgitation with a partially flail anterior mitral valve leaflet and prolapse of the posterior mitral valve leaflet (Figure 3). A transesophageal echocardiogram showed a small linear echodensity on the aortic valve, anteriorly directed mitral regurgitation, and linear mobile densities attached to the anterior mitral annulus measuring up to 1.6 cm, concerning for endocarditis (Figure 4). 

**Figure 5:**
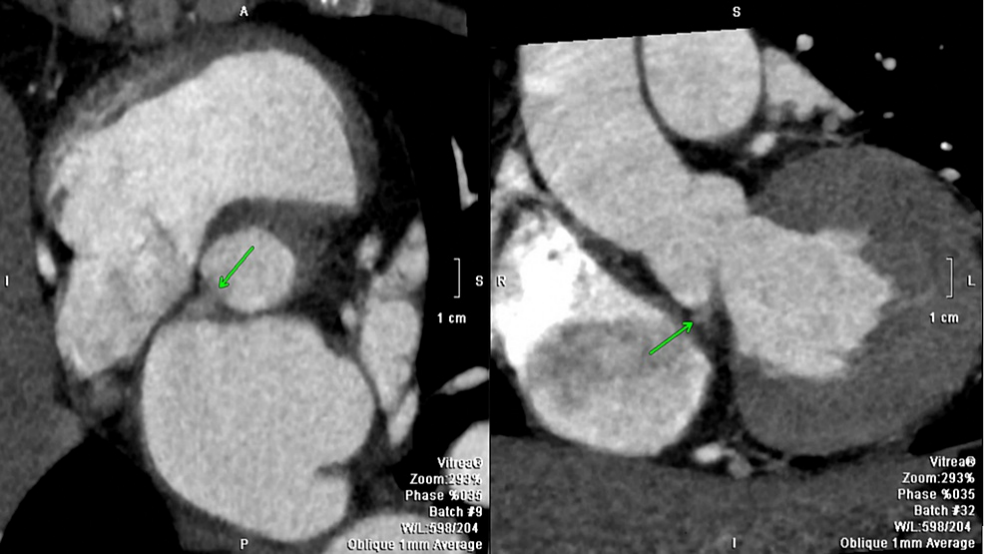
CT cardiac structure and morphology with contrast. A small collection of contrast is visualized just below the non-coronary cusp of the aortic valve associated with a defect at the annulus. The collection extends into the interatrial septum. A possible communication between the collection and the left atrium is also seen.

MRI of the brain showed several small foci of acute infarction in the left caudate head and bilateral frontal lobes, possibly septic emboli. The patient's neurologic examination remained unremarkable, the National Institutes of Health Stroke Scale was 0, and given uncertain time of onset, the patient was deemed not a candidate for thrombectomy. The otolaryngology was consulted for the oral mass, and CT of the neck with contrast was ordered, which showed a 2.0 x 1.5 x 0.5 cm rim-enhancing low-attenuation structure in the right floor of the mouth adjacent to the mandible without bony erosion. It was suspected that there may be possible sialadenitis vs malignancy, and an outpatient biopsy of the mass was recommended for further workup once the inflammation subsides. Cardiothoracic surgery was consulted and planned for mitral valve replacement with possible aortic valve replacement and left atrial appendage clipping. Cardiology was consulted for left and right heart catheterization prior to the valve replacement, and it showed moderate pulmonary artery hypertension in the setting of elevated wedge pressure to 28 mmHg and also mild, non-flow-limiting coronary artery disease. CT cardiac structure and morphology with contrast was then obtained, which showed a small defect at the annulus immediately below the non-coronary cusp extending into the collection of contrast, which eroded into the interatrial septum immediately below the aortic root, likely representing an aortic root abscess (Figure 5). 

Given the complex nature of the patient’s pathology, a multidisciplinary team consisting of infectious disease, orthopedic surgery, otolaryngology, cardiothoracic surgery, interventional cardiology, cardiology, and neurology was assembled to guide the patient’s care. The patient was continued on vancomycin, ceftriaxone, and metronidazole. The patient’s back pain improved on subsequent days and she remained neurologically stable throughout her hospital course; no surgical intervention was required for her spinal infection. The imaging findings of severe mitral regurgitation with flail P3, aortic and mitral valve vegetations, and the interatrial septal abscess necessitated surgical intervention. The patient subsequently underwent aortic valve resection with bioprosthetic aortic valve replacement, mitral valve resection with bioprosthetic mitral valve replacement, clipping of the left atrial appendage, and unroofing, obliteration, and pericardial patching of the intra-atrial abscess. The abscess was located in the left atrial wall above the A2 annulus and communicated with the left ventricular outflow tract (LVOT) just below the aortic annulus of the non-coronary cusp. The aortic valve did not appear to be involved intraoperatively but was excised and replaced in order to allow for adequate obliteration of the abscess cavity. 

The patient was started on aspirin and apixaban for her bioprosthetic valve replacements. Infectious disease recommended a six-week course of IV ceftriaxone, followed by suppression with oral antibiotics indefinitely. Three months after discharge from the hospital, the patient continued to do well without evidence of postoperative complications. She completed the six-week course of IV antibiotics and was transitioned to oral cefdinir therapy. The patient remained neurologically intact and continued to follow up with the multidisciplinary teams.

## Discussion

IE is a complex disorder that often requires management by an allied team of providers from different areas of expertise. In a large prospective cohort study by Murdoch et al., 72.1 % of cases of IE were shown to have native valve endocarditis with mitral (41.1%) and aortic (37.6%) valves being involved most frequently, as in the case of our patient [2]. Little information is available, however, about multivalvular IE and it is considered much rarer than single-valvular IE. The incidence of interseptal cardiac abscess in IE is also unknown and sporadic case reports form the base of our current understanding of the rare IE cases. In a single-institution, retrospective chart review study by Kim et al., multivalvular IE was recognized as a separate clinical entity and it was shown that the mortality of multivalvular IE is higher than that of single-valvular IE (21% vs 18%, respectively) [3]. Furthermore, multivalvular IE patients were found to have an increased incidence of complications including congestive heart failure and acute renal failure [3]. 

Common complications of IE include stroke, embolization to other organs, valvular vegetations, intracardiac abscess, and periannular extension of the infection. Intracardiac abscesses often result from the extension of the infection of the cardiac valves, with the highest predisposition to the aortic valve [4]. Moreover, those with prosthetic valves are at a higher risk of developing perivalvular abscess. In patients with native valves, bacterial endocarditis is more likely to occur on previously damaged valves [4]. From this perspective, our patient’s case was unique in that she had a native valve infection with no known risk factors, and the abscess was located within the left atrial wall. During the surgical exploration, the interatrial abscess was seen from the left atrial wall above the A2 annulus, which communicated with the LVOT below the aortic annulus of the non-coronary cusp, and, subsequently, a transseptal, transaortic obliteration of the abscess was performed. 

In most cases of IE, the mainstay of treatment is a prolonged IV antibiotic therapy for up to six weeks targeted to the organism isolated in the blood culture [5,6]. Surgical intervention is warranted in certain cases to prevent serious complications, such as progression of heart failure, irreversible structural damage, and systemic embolism. Current indications for surgical intervention include 1) valvular dysfunction, usually aortic or mitral regurgitation, causing heart failure; 2) perivalvular extension of infection with development of an abscess, fistula, and/or heart block; 3) infection with a difficult-to-treat pathogen, such as fungal or multidrug-resistant organisms; and 4) persistent bacteremia or fever despite appropriate antibiotic therapy for greater than seven days [5,7]. Many studies have shown that a vegetation size >10 mm is associated with increased embolic events and mortality [8,9]. Our patient met multiple criteria including severe mitral regurgitation, perivalvular abscess and fistula, and vegetation greater than 10 mm in size, and thus was managed with a surgical intervention and long-term IV antibiotics with a successful outcome.

## Conclusions

IE is a complex condition with a broad array of clinical manifestations and systemic complications. Multivalvular IE with interseptal abscess is considered a rare entity, and studies have shown an increased risk of complications and mortality in these cases. Here, we present a case of an IE that involved both mitral and aortic valves as well as an interatrial septal abscess that required extensive surgical interventions including aortic and mitral valve resection with bioprosthetic valve replacements and a pericardial obliteration and patching of the intra-atrial abscess. Effective management of IE involves a multidisciplinary approach with careful surgical planning, medication management, and close follow-ups.
